# Urinary Biomarkers of Kidney Tubule Health and Mortality in Persons with CKD and Diabetes Mellitus

**DOI:** 10.34067/KID.0000000000000226

**Published:** 2023-08-03

**Authors:** George Vasquez-Rios, Ronit Katz, Emily B. Levitan, Mary Cushman, Chirag R. Parikh, Paul L. Kimmel, Joseph V. Bonventre, Sushrut S. Waikar, Sarah J. Schrauben, Jason H. Greenberg, Mark J. Sarnak, Joachim H. Ix, Michael G. Shlipak, Orlando M. Gutierrez

**Affiliations:** 1 Division of Nephrology, Department of Internal Medicine, Icahn School of Medicine at Mount Sinai, Manhattan, New York; 2 Department of Obstetrics and Gynecology, University of Washington, Seattle, Washington; 3 Department of Epidemiology, University of Alabama at Birmingham, Birmingham, Alabama; 4 Departments of Medicine and Pathology and Laboratory Medicine, Larner College of Medicine at the University of Vermont, Burlington, Vermont; 5 Section of Nephrology, Department of Internal Medicine, Johns Hopkins School of Medicine, Baltimore, Maryland; 6 National Institute of Diabetes and Digestive and Kidney Diseases, Bethesda, Maryland; 7 Division of Nephrology, Department of Medicine, Brigham and Women's Hospital, Boston, Massachusetts; 8 Section of Nephrology, Department of Medicine, Boston Medical Center, Boston, Massachusetts; 9 Department of Medicine, Perelman School of Medicine, Center for Clinical Epidemiology and Biostatistics at the Perelman School of Medicine at the University of Pennsylvania, Philadelphia, Pennsylvania; 10 Section of Nephrology, Department of Pediatrics, Program of Applied Translational Research, Yale University School of Medicine, New Haven, Connecticut; 11 Division of Nephrology, Department of Medicine, Tufts Medical Center, Boston, Massachusetts; 12 Division of Nephrology-Hypertension, Department of Medicine, University of California San Diego, San Diego, California; 13 Veterans Affairs San Diego Healthcare System, San Diego, California; 14 Kidney Health Research Collaborative, San Francisco Veterans Affairs Healthcare System and University of California, San Francisco, California; 15 Departments of Medicine and Epidemiology, University of Alabama at Birmingham, Birmingham, Alabama

**Keywords:** chronic kidney disease, diabetes mellitus, kidney tubule, mortality

## Abstract

**Key Points:**

- Among adults with diabetes and CKD, biomarkers of kidney tubule health were associated with a greater risk of death, independent of eGFR, albuminuria, and additional risk factors.
- Higher urine levels of YKL-40 and KIM-1 were associated with a greater risk of death. For cause-specific death, UMOD was independently and inversely associated with the risk of cardiovascular death.

**Background:**

Kidney disease assessed by serum creatinine and albuminuria are strongly associated with mortality in diabetes. These markers primarily reflect glomerular function and injury. Urine biomarkers of kidney tubule health were recently associated with the risk of kidney failure in persons with CKD and diabetes. Associations of these biomarkers with risk of death are poorly understood.

**Methods:**

In 560 persons with diabetes and eGFR ≤60 ml/min per 1.73 m^2^ from the Reasons for Geographic and Racial Differences in Stroke study (47% male, 53% Black), we measured urine biomarkers of kidney tubule health at baseline: monocyte chemoattractant protein-1 (MCP-1), alpha-1-microglobulin, kidney injury molecule-1 (KIM-1), EGF, chitinase-3-like protein 1 (YKL-40), and uromodulin (UMOD). Cox proportional hazards regression was used to examine the associations of urine biomarkers with all-cause and cause-specific mortality in nested models adjusted for urine creatinine, demographics, mortality risk factors, eGFR, and urine albumin.

**Results:**

The mean (SD) age was 70 (9.6) years, and baseline eGFR was 40 (3) ml/min per 1.73 m^2^. There were 310 deaths over a mean follow-up of 6.5 (3.2) years. In fully adjusted models, each two-fold higher urine concentration of KIM-1 and YKL-40 were associated with all-cause mortality (hazard ratio [HR] 1.15, 95% confidence interval [CI], 1.01 to 1.31 and 1.13, 95% CI, 1.07 to 1.20, respectively). When examining cause-specific mortality, higher UMOD was associated with a lower risk of cardiovascular death (adjusted HR per two-fold higher concentration 0.87, 95% CI, 0.77 to 0.99), and higher MCP-1 was associated with higher risk of cancer death (HR per two-fold higher concentration 1.52, 95% CI, 1.05 to 2.18).

**Conclusion:**

Among persons with diabetes and CKD, higher urine KIM-1 and YKL-40 were associated with a higher risk of all-cause mortality independently of established risk factors. Urine UMOD and MCP-1 were associated with cardiovascular and cancer-related death, respectively.

## Graphical abstract

**Figure:**
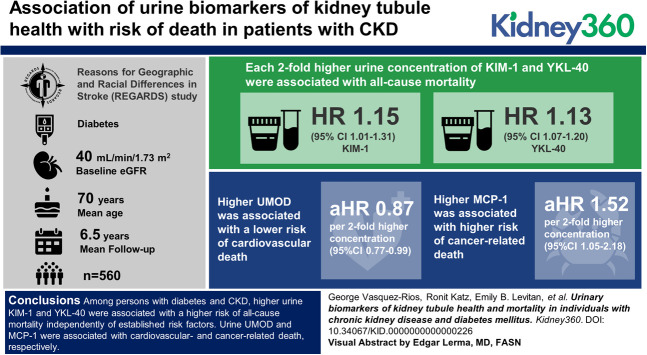

## Introduction

CKD is a robust risk factor of death in persons with diabetes. Most literature examining the association of CKD with mortality has focused on creatinine- or cystatin C–based eGFR and albuminuria.^1^ However, eGFR and urine albumin excretion largely reflect glomerular filtration or injury and incompletely capture the health of other critical components of the nephron, such as tubules and the interstitium.^2–4^ Prior studies showed that biomarkers of kidney tubular injury and dysfunction are associated with kidney disease development or progression above and beyond eGFR and albuminuria, contributing to our understanding of biological pathways implicated in CKD.^5,6^

We recently reported that urine biomarkers of kidney tubule health (monocyte chemoattractant protein-1 [MCP-1], alpha-1-microglobulin [*α*1m], kidney injury molecule-1 [KIM-1], EGF, chitinase-3-like protein 1 [YKL-40], uromodulin [UMOD]) were differentially associated with the risk of kidney failure requiring replacement therapy in persons with diabetes and an eGFR of <60 ml/min per 1.73 m^2^.^7^ Markers of tubule-interstitial injury and fibrosis may also carry important prognostic information for death.^2–4,8–11^ Higher circulating levels of MCP-1 and YLK-40 were observed in persons who developed cardiovascular events and progressive heart disease as compared with persons with lower levels.^12,13^ Higher urine *α*1m was independently associated with hypertension, cardiovascular disease, and death in prospective studies of persons with AKI and CKD without diabetes.^14,15^ The associations of urine KIM-1, EGF, and UMOD with death have been studied less, particularly in high-risk populations, such as those with diabetes mellitus. Therefore, the goal of this study was to assess the association between urine biomarkers of kidney tubule health with all-cause and cause-specific mortality in persons with diabetes mellitus and eGFR <60 ml/min per 1.73 m^2^ from the Reasons for Geographic and Racial Differences in Stroke (REGARDS) study.

## Methods

The REGARDS study is a prospective cohort study designed to ascertain reasons for excessive stroke risk and stroke mortality in persons living in the Southeastern United States and Black adults. Details of the study design were published elsewhere.^16^ Briefly, a total of 30,239 Black and White persons (55% women) at least 45 years were enrolled between January 2003 and October 2007. Persons information (sociodemographic characteristics, cardiovascular risk factors, and use of medications) was initially collected through computer-assisted telephone interview, and then, trained personnel conducted an in-home visit to obtain anthropometric measures, for physical assessment, to collect blood and urine specimens, and to obtain data on medication inventory.^17^ The REGARDS protocol was approved by the institutional review boards at the participating centers, and all persons provided written informed consent. This study’s aims, approach, and analysis plan were planned *a priori* and reviewed and approved by the REGARDS publications committee, as was the final article, including assessment of adherence to the plan.

### Primary Exposures

Urine KIM-1, MCP-1, YKL-40, and EGF were measured on the Luminex platform with a laboratory-developed multiplex assay (Luminex Corporation, Austin, TX). UMOD was measured on the MSD R-PLEX (Meso Scale Diagnostics, LLC., Rockville, MD). Urine *α*1m was measured on a Siemens BNII nephelometer (Siemens, Inc., Munich, Germany). Urine aliquots were kept in continuous laboratory storage at −80°C until they were thawed for biomarker measurements. Personnel conducting biomarker measurements were blinded to clinical outcomes. All measurements, except *α*1m, were made in duplicate, and mean values were used in analyses. If the intra-assay coefficient of variation exceeded 15%, the assay was repeated.

### Outcomes

The primary outcome of this study was all-cause mortality, and the secondary outcome was cause-specific mortality. Death was detected through active surveillance and ascertained in three ways: (*1*) during the 6-month follow-up phone calls or when study personnel were informed of the death by the persons relative or proxy, (*2*) through the Social Security Death Index or National Death Index, and (*3*) other death events were identified through searches of the Social Security Administration's Master Death File. The specific cause of death was obtained from interview with the proxy, from death certificates, and by clinician evaluators of medical records. Details about the methods were published elsewhere.^18^ Cause-specific mortality was categorized as cardiovascular death (myocardial infarction, stroke, sudden cardiac death, congestive heart failure, not cardiac but other cardiovascular cause), cancer death, and others (accident or suicide, other noncardiac and nonstroke causes adjudicated by the investigator or unclassifiable). Further details on the process of identification of the cause of death have been published elsewhere.^19^ All-cause death and cause-specific death through December 31, 2014, were available for the current analyses.

### Covariates

Demographic and clinical characteristics, including age, sex, race, income, education, smoking status, and alcohol use, were determined by self-report. Height and weight were measured during the in-person visit to estimate the body mass index. Hypertension was defined when an average of two seated BP readings had a systolic BP of ≥140 mm Hg and/or diastolic BP of ≥90 mm Hg or when the person reported use of antihypertensive medications. Diabetes was defined as self-reported use of insulin or oral hypoglycemic agents, fasting glucose ≥126 mg/dl, or random glucose ≥200 mg/dl. History of coronary heart disease (CHD) was defined as having any of the following: evidence of myocardial infarction on the baseline electrocardiograph, self-report of a history of a cardiac procedure (coronary artery bypass surgery or percutaneous coronary intervention), or self-reported history of myocardial infarction. History of stroke was determined by self-report as well. A suspected history of heart failure at baseline was determined by current use of heart failure–related medications at the baseline visit. The lipid profile, including high-density lipoprotein and triglycerides, was measured from fasting samples. High-sensitivity C-reactive protein was measured using a high-sensitivity particle–enhanced immunonephelometric assay. Serum creatinine was calibrated to an international isotope dilution mass spectroscopic-traceable standard, measured by colorimetric reflectance spectrophotometry, and eGFR was calculated using the 2009 Chronic Kidney Disease Epidemiology Collaboration equation.^20^ Albumin and creatinine were measured using the random spot urine specimen by nephelometry (BN ProSpec Nephelometer, Dade Behring, Marburg, Germany) and Modular-P chemistry analyzer (Roche/Hitachi, Indianapolis, IN), respectively.

### Derivation of the Study Population

Of persons with diabetes and an eGFR of <60 ml/min per 1.73 m^2^ who did not have prevalent kidney failure requiring replacement therapy at the baseline visit (*n*=1092), we randomly selected 600 persons from this cohort study without regard to biomarker status or mortality outcomes. Forty persons did not have an adequate volume of stored urine for biomarker measurement, resulting in 560 persons in the final analyzed sample.

### Statistical Analyses

Descriptive statistics were used to compare person characteristics across quartiles of each urine biomarker. After confirming the assumption of the proportionality of hazards, Cox regression models were used to estimate the hazard ratios (HRs) for all-cause and cause-specific death as a function of each two-fold higher concentration of a biomarker, considered individually. Model 1 was adjusted for age, sex, race, education, and urine creatinine. Model 2 was further adjusted for systolic BP, use of hypertension meds, body mass index, smoking, history of CHD, history of stroke, C-reactive protein, eGFR, and urine albumin, to determine the added information obtained above and beyond standard clinical markers of glomerular health. In all models, urine biomarkers were analyzed on a continuous scale after log base 2 transformation (interpreted as per two-fold higher for each biomarker) and in quartiles, with the lowest quartile serving as the referent group. Values of *α*1m that were below the lower limit of detection (5.47 mg/L, *n*=88, 14%) were set to 5.47 mg/L (no other biomarkers had values below the lower limit). We examined for effect modification by race and sex by testing the statistical significance of a multiplicative interaction term in fully adjusted models, for each biomarker separately. A two-tailed *P* value of <0.05 was considered statistically significant for all analyses. All analyses were conducted using SPSS version 26.0 (IBM Corp., Armonk, NY) and R version 4.0.2 (R Foundation for Statistical Computing, Vienna, Austria).

## Results

Among the 560 persons included in these analyses, 47% were male, 53% were Black, the baseline mean (SD) age was 70 (10) years, mean eGFR was 40 (3) ml/min per 1.73 m^2^, and the median (interquartile range) albumin-to-creatinine ratio was 33 (10,213) mg/g. Other demographic and clinical characteristics of the study sample are presented by quartiles of urine YKL-40 in Table 1. Persons in the highest quartiles of YKL-40 were less likely to be of Black race, had lower education, were less likely to have a history of heart failure, and were more likely to have a history of CHD and stroke and to have higher urine albumin-to-creatinine ratios. Baseline characteristics across biomarker quartiles largely aligned with YKL-40 and are presented in Supplemental Tables 1–5.

**Table 1:** Baseline characteristics of the analytical cohort on the basis of chitinase-3-like protein 1 quartiles in the Reasons for Geographic and Racial Differences in Stroke subcohort

YKL-40 (pg/ml)				
Quartile (Range)	Q1 (<169)	Q2 140 (169–416)	Q3 140 (417–958)	Q4 140 (>958)
*N*	140	140	140	140
Age, yr (SD)	69 (8)	71 (9)	71 (8)	70 (9)
Male sex (%)	72 (52)	73 (52)	71 (51)	46 (33)
Black Race (%)	67 (48)	68 (49)	80 (57)	84 (60)
**Education (%)**				
Less than high school	23 (16)	28 (20)	36 (26)	36 (26)
High school graduate	42 (30)	31 (22)	39 (28)	40 (29)
Some college	34 (24)	34 (24)	38 (27)	37 (26)
College graduate and above	41 (29)	47 (34)	27 (19)	27 (19)
Insured	136 (97)	137 (98)	133 (95)	136 (97)
BMI	32.3 (6.6)	31.3 (6.6)	32.2 (6.8)	31.6 (6.3)
Hypertension (%)	120 (86)	126 (90)	122 (87)	123 (88)
SBP, mm Hg (SD)	131 (18)	130 (17)	133 (16)	137 (22)
DBP, mm Hg (SD)	74 (10)	73 (11)	73 (11)	77 (12)
Heart failure	57 (41)	56 (40)	54 (39)	65 (46)
CAD	53 (38)	60 (43)	51 (36)	61 (44)
Stroke	24 (17)	13 (9)	24 (17)	27 (19)
**Smoking**				
Never	69 (49)	56 (40)	58 (41)	63 (45)
Former	59 (42)	72 (51)	71 (51)	59 (42)
Current	12 (9)	12 (9)	11 (8)	18 (13)
Antihypertensive use	113 (81)	121 (86)	117 (84)	118 (84)
ACE inhibitor/ARB use	110 (79)	111 (79)	99 (71)	95 (68)
Diuretic use	97 (69)	96 (69)	97 (69)	87 (62)
eGFR, ml/min per 1.73 m^2^	43 (12)	44 (12)	41 (12)	35 (13)
**UACR, mg/g**	24 (7, 87)	27 (9, 66)	22 (9, 150)	224 (25, 1551)
<30	74 (53)	76 (54)	81 (58)	39 (28)
30–300	52 (37)	47 (34)	29 (21)	36 (26)
≥300	14 (10)	17 (12)	30 (21)	65 (46)

YKL-40, chitinase-3-like protein 1; BMI, body mass index; SBP, systolic BP; DBP, diastolic BP; CAD, coronary artery disease; ACE, angiotensin-converting enzyme; ARB, angiotensin receptor II blocker; UACR, urine albumin–creatinine ratio.

### Urinary Biomarkers' Association with All-Cause Death

**Figure 1:**
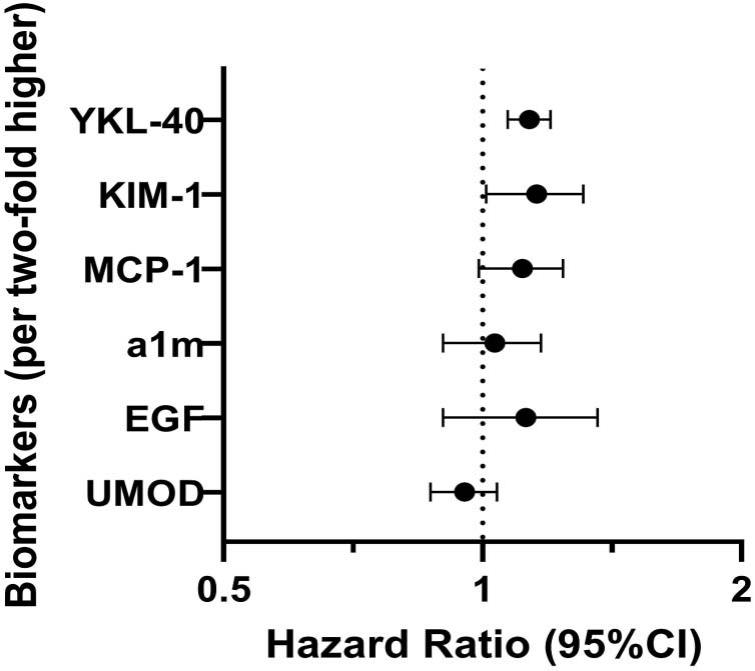
**Forest plot analysis showing association between biomarkers and all-cause death and their respective adjusted hazard ratios (95% CI).** Adjusted hazard ratios (fully adjusted models): adjusted for age, sex, race, education, and urine creatinine, systolic BP, use of hypertension meds, body mass index, smoking, history of coronary heart disease, history of stroke, C-reactive protein, eGFR, and urine albumin. *α*1m, alpha-1-microglobulin; CI, confidence interval; KIM-1, kidney injury molecule-1; MCP-1, monocyte chemoattractant protein-1; UMOD, uromodulin; YKL-40, chitinase-3-like protein 1.

A total of 310 persons died over a mean (SD) follow-up of 6.5 (3.2) years, reflecting an annual mortality rate of 8.6%. Table 2 summarizes multivariable-adjusted associations of urine biomarkers with all-cause death. Higher concentrations of YKL-40, KIM-1, MCP-1, and *α*1m were associated with higher risk of death in models adjusted for age, race, sex, education, and urine creatinine. By contrast, higher concentrations of UMOD and EGF were associated with lower risk of all-cause mortality. However, after full adjustment, including eGFR and urine albumin, only YKL-40 (HR 1.13, 95% confidence interval, 1.07 to 1.20) and KIM-1 (HR 1.15, 95% confidence interval, 1.01 to 1.31) remained independently associated with all-cause mortality (Figure 1). None of the associations were modified by sex or race (all *P* interactions > 0.05). When biomarkers were modeled as quartiles, none of the biomarkers demonstrated trends in association with mortality compared with the reference quartile, except for YKL-40. Persons in the highest quartile of YKL-40 had an approximately 50% higher mortality risk compared with persons in the lowest quartile.

**Table 2:** Association of baseline urine biomarkers with all-cause mortality

Biomarker	Range (Min, Max)	Mortality Rate (%/yr)	Model 1^a^	Model 2^b^
			HR (95% CI)	HR (95% CI)
*α*1m (mg/L)	Continuous (per two-fold higher)	8.61	1.31 (1.20 to 1.44)	1.03 (0.90 to 1.17)
	Q1 (<8.25)	6.90	1.00	1.00
	Q2 (8.25–16.60)	7.65	1.17 (0.82 to 1.66)	1.01 (0.71 to 1.46)
	Q3 (16.61–32.10)	9.06	1.60 (1.12 to 2.27)	0.97 (0.66 to 1.43)
	Q4 (>32.10)	11.16	2.07 (1.47 to 2.91)	0.85 (0.54 to 1.34)
EGF (pg/ml)	Continuous (per two-fold higher)	8.61	0.75 (0.63 to 0.89)	1.11 (0.90 to 1.36)
	Q1 (<767)	12.15	1.00	1.00
	Q2 (767–1017)	9.56	0.77 (0.57 to 1.04)	1.18 (0.84 to 1.65)
	Q3 (1018–1358)	7.15	0.71 (0.51 to 0.99)	1.25 (0.86 to 1.83)
	Q4 (>1358)	6.30	0.58 (0.40 to 0.85)	1.28 (0.83 to 1.98)
KIM-1 (pg/ml)	Continuous (per two-fold higher)	8.61	1.36 (1.23 to 1.51)	1.15 (1.01 to 1.31)^c^
	Q1 (<1016)	7.80	1.00	1.00
	Q2 (1016–1775)	8.38	1.46 (1.04 to 2.06)	1.14 (0.80 to 1.63)
	Q3 (1776–3464)	8.53	1.47 (1.05 to 2.05)	0.97 (0.68 to 1.41)
	Q4 (>3465)	9.78	2.20 (1.52 to 3.19)	1.14 (0.74 to 1.75)
MCP-1 (pg/ml)	Continuous (per two-fold higher)	8.61	1.27 (1.16 to 1.40)	1.11 (0.99 to 1.24)
	Q1 (<130)	8.82	1.00	1.00
	Q2 (130–216)	6.88	0.94 (0.66 to 1.33)	0.72 (0.50 to 1.04)
	Q3 (217–385)	8.26	1.32 (0.93 to 1.87)	1.03 (0.72 to 1.47)
	Q4 (>385)	10.85	1.87 (1.31 to 2.68)	1.13 (0.76 to 1.67)
UMOD (μg/ml)	Continuous (per two-fold higher)	8.61	0.86 (0.80 to 0.94)	0.95 (0.87 to 1.04)
	Q1 (<3080904)	11.80	1.00	1.00
	Q2 (3080904–6197501)	8.61	0.74 (0.55 to 1.01)	0.94 (0.68 to 1.30)
	Q3 (6197502–11294503)	7.20	0.60 (0.44 to 0.83)	0.80 (0.57 to 1.13)
	Q4 (>11294503)	7.30	0.59 (0.42 to 0.83)	0.87 (0.61 to 1.26)
YKL-40 (pg/ml)	Continuous (per two-fold higher)	8.61	1.22 (1.16 to 1.28)	1.13 (1.07 to 1.20)^c^
	Q1 (<169)	6.92	1.00	1.00
	Q2 (169–416)	8.20	1.22 (0.86 to 1.72)	1.03 (0.72 to 1.46)
	Q3 (417–958)	7.67	1.22 (0.87 to 1.72)	1.01 (0.71 to 1.43)
	Q4 (>958)	12.35	2.61 (1.87 to 3.65)	1.58 (1.15 to 2.45)^c^

HR, hazard ratio; CI, confidence interval; *α*1m, alpha-1-microglobulin; KIM-1, kidney injury molecule-1; MCP-1, monocyte chemoattractant protein-1; UMOD, uromodulin; YKL-40, chitinase-3-like protein 1.

a Model 1: adjusted for age, sex, race, education, and urine creatinine.

b Model 2: further adjusted for systolic BP, use of hypertension meds, BMI, smoking, history of CHD, history of stroke, CRP, eGFR, and urine albumin.

c Statistically significant (*p* < 0.05).

### Urinary Biomarkers and Cause-Specific Death

We next evaluated associations of each biomarker with cause-specific death (Table 3). Urine UMOD was the only biomarker independently associated with *cardiovascular death* in fully adjusted models; higher UMOD associated with lower cardiovascular death risk. Higher MCP-1 was associated with greater risk of *cancer death* in fully adjusted models. Higher YKL-40, KIM-1, EGF, and MCP-1 were associated with higher risk of *other death* in fully adjusted models.

**Table 3:** Association of baseline urine biomarkers with cause-specific mortality in the Reasons for Geographic and Racial Differences in Stroke subcohort

Biomarker (log2-Transformed)	No. of Deaths	Mortality Rate (%/yr)	Model 1^a^	Model 2^b^
			HR (95% CI)	HR (95% CI)
CVD Death	121	3.36		
*α*1m			1.23 (1.07 to 1.43)	0.97 (0.79 to 1.20)
EGF			0.73 (0.55 to 0.96)	0.92 (0.66 to 1.28)
KIM-1			1.22 (1.04 to 1.42)	0.97 (0.79 to 1.19)
MCP-1			1.29 (1.11 to 1.50)	1.11 (0.92 to 1.34)
UMOD			0.83 (0.74 to 0.93)	0.87 (0.77 to 0.99)^c^
YKL-40			1.18 (1.09 to 1.28)	1.07 (0.97 to 1.17)
Cancer Death	30	0.83		
*α*1m			1.24 (0.92 to 1.66)	1.12 (0.72 to 1.74)
EGF			0.63 (0.35 to 1.11)	0.73 (0.35 to 1.50)
KIM-1			1.36 (0.98 to 1.89)	1.48 (0.95 to 2.30)
MCP-1			1.39 (1.02 to 1.91)	1.52 (1.05 to 2.18)^c^
UMOD			0.92 (0.70 to 1.20)	0.98 (0.75 to 1.29)
YKL-40			1.11 (0.94 to 1.32)	1.12 (0.92 to 1.35)
Other Death	159	4.42		
*α*1m			1.39 (1.22 to 1.58)	1.03 (0.86 to 1.24)
EGF			0.79 (0.62 to 1.01)	1.38 (1.05 to 1.82)^c^
KIM-1			1.49 (1.29 to 1.71)	1.25 (1.04 to 1.50)^c^
MCP-1			1.24 (1.08 to 1.43)	1.04 (0.88 to 1.23)
UMOD			0.89 (0.79 to 1.00)	1.04 (0.91 to 1.18)
YKL-40			1.27 (1.18 to 1.36)	1.18 (1.08 to 1.29)^c^

HR, hazard ratio; CI, confidence interval; CVD, cardiovascular disease; *α*1m, alpha-1-microglobulin; KIM-1, kidney injury molecule-1; MCP-1, monocyte chemoattractant protein-1; UMOD, uromodulin; YKL-40, chitinase-3-like protein 1.

a Model 1: adjusted for age, sex, race, education, and urine creatinine.

b Model 2: further adjusted for systolic BP, use of hypertension meds, BMI, smoking, history of CHD, history of stroke, CRP, eGFR, and urine albumin.

c Statistically significant (*p* < 0.05).

## Discussion

In this study of adults with diabetes and CKD, we demonstrated that several biomarkers reflecting kidney tubule health were associated with greater risk of death, independent of demographics, risk factors, and eGFR and albuminuria. Specifically, higher urine YKL-40 and KIM-1 were associated with greater risk of all-cause death. For cause-specific death, UMOD was independently and inversely associated with risk of cardiovascular death while higher MCP-1 was associated with higher risk of cancer-related death in fully adjusted models.

There are scant clinical data examining the association of urine YKL-40 with death among persons with CKD and diabetes. In an analysis from Systolic Blood Pressure Intervention Trial that focused on hypertensive persons with eGFR <60 ml/min per 1.73 m^2^ and without diabetes mellitus, urine YKL-40 was associated with a higher risk of death. Nonetheless, it was not associated with cardiovascular mortality in fully adjusted models.^8^ Diabetes was an exclusion criterion for Systolic Blood Pressure Intervention Trial, so the present findings add to the literature. Our findings further support the role of interstitial inflammation and fibrosis as independent risk factors of death in persons with diabetes and CKD.^21,22^ Two large prospective studies consisting primarily of persons with stable coronary artery disease found that YKL-40 was independently associated with cardiovascular complications and death.^23,24^ Although we did not observe an independent association between YKL-40 and cardiovascular death in this study, we interpret these findings with caution because our sample size for cause-specific mortality end points was smaller, reducing our power to detect modest associations.^23–25^

The findings that higher urine KIM-1 is independently associated with higher risk of all-cause death in those with diabetes and CKD are novel and underscore the role of kidney tubule health in the pathophysiology of death among high-risk persons. Previous reports reported an independent association between urine KIM-1 and all-cause death in community-dwelling persons with and without baseline CKD.^10,26,27^ urine KIM-1 was also associated with adverse heart failure outcomes and longitudinal kidney function decline among persons with and without diabetes mellitus in some prospective studies.^27–31^ However, few studies examined these associations in specific high-risk populations, such as those with diabetes mellitus and eGFR <60 ml/min per 1.73 m^2^ or that included a large proportion of Black persons.

We found that higher urine UMOD was independently associated with lower risk of cardiovascular death in person with diabetes and CKD. These data add to previous literature, which consistently show that UMOD is inversely associated with outcomes, including CKD progression, cardiovascular disease, and mortality in persons with and without CKD.^15,32,33^ Furthermore, our results expand prior genome-wide association studies that suggest that UMOD variants may be protective against diabetic nephropathy susceptibility.^34^ Although the reasons for this relationship are unclear, it is hypothesized that UMOD reflects the distal tubule's protein synthetic capacity and protects against infection and injury. In addition, urine MCP-1 was independently associated with cancer-related death, although the number of events for this analysis was low. Finally, we note that EGF, KIM-1, and YKL-40 were independently associated with other (noncardiovascular and noncancer) deaths, potentially arguing for pathological mechanisms that could affect distant organs other than the kidney and heart/vasculature. The nature of these associations warrants further evaluation because EGF has been implicated in other conditions, including infections and mortality, elsewhere.

This study had several strengths, including a relatively large sample of community-dwelling persons with both diabetes and CKD and well-ascertained baseline clinical data and outcomes. The availability of multiple biomarkers concurrently provided opportunities to examine and compare strengths of associations for the different death outcomes across biomarkers. All biomarkers were measured using baseline samples in the same central laboratory in a protocolized fashion with detailed control measures. Furthermore, by design, we include a large proportion of Black persons who are known to be at excess risk of death. This study also had limitations, including biovariability of the tubule biomarkers and lack of follow-up measures of urine biomarkers and clinical data. These factors would tend to bias results to the null hypothesis. All measurements, except *α*1m, were made in duplicate, which may have affected the precision of *α*1m measurements. We did not account for hospitalizations or emergency department visits as well as additional insults that could have played a role in the overall risk of death, potentially affecting the internal validity.

In conclusion, urine YKL-40 and KIM-1 were associated with the risk of all-cause death among persons with diabetes and CKD, independent of eGFR, albuminuria, and other risk factors. In addition, UMOD and MCP-1 were associated with cardiovascular and cancer-related deaths, respectively. These findings may aid in the elucidation of potential pathways within kidney disease driving different risks of death.

## Supplementary Material

## Supplemental Material

Supplemental Table 1. Baseline characteristics of the analytical cohort on the basis of KIM-1 quartiles in the REGARDS subcohort.

Supplemental Table 2. Baseline characteristics of the analytical cohort on the basis of MCP-1 quartiles in the REGARDS subcohort.

Supplemental Table 3. Baseline characteristics of the analytical cohort on the basis of EGF quartiles in the REGARDS subcohort.

Supplemental Table 4. Baseline characteristics of the analytical cohort on the basis of UMOD quartiles in the REGARDS subcohort.

Supplemental Table 5. Baseline characteristics of the analytical cohort on the basis of a1m quartiles in the REGARDS subcohort.

## References

[1] Afkarian M Sachs MC Kestenbaum B, . Kidney disease and increased mortality risk in type 2 diabetes. J Am Soc Nephrol. 2013;24(2):302–308. doi:10.1681/ASN.2012070718 23362314 PMC3559486

[2] Howie AJ Ferreira MA Adu D. Prognostic value of simple measurement of chronic damage in renal biopsy specimens. Nephrol Dial Transplant. 2001;16(6):1163–1169. doi:10.1093/ndt/16.6.1163 11390715

[3] Takebayashi S Kiyoshi Y Hisano S, . Benign nephrosclerosis: incidence, morphology and prognosis. Clin Nephrol. 2001;55(5):349–356.PMID: 11393379. 11393379

[4] Nath KA. Tubulointerstitial changes as a major determinant in the progression of renal damage. Am J Kidney Dis. 1992;20(1):1–17. doi:10.1016/s0272-6386(12)80312-x 1621674

[5] Ix JH Shlipak MG. The promise of tubule biomarkers in kidney disease: a review. Am J Kidney Dis. 2021;78(5):719–727. doi:10.1053/j.ajkd.2021.03.026 34051308 PMC8545710

[6] Lopez-Giacoman S Madero M. Biomarkers in chronic kidney disease, from kidney function to kidney damage. World J Nephrol. 2015;4(1):57–73. doi:10.5527/wjn.v4.i1.57 25664247 PMC4317628

[7] Sarnak MJ Ronit K JoachimIx H, . Plasma biomarkers as risk factors for incident chronic kidney disease. Kidney Int Rep. 2022;7(7):1493–1501. doi:10.1016/j.ekir.2022.03.018 35812266 PMC9263237

[8] Jotwani VK Lee A Estrella M, . Urinary biomarkers of tubular damage are associated with mortality but not cardiovascular risk among systolic blood pressure intervention trial participants with chronic kidney disease. Am J Nephrol. 2019;49(5):346–355. doi:10.1159/000499531 30939472 PMC6491265

[9] Coca SG Garg AX Thiessen-Philbrook H, . Urinary biomarkers of AKI and mortality 3 years after cardiac surgery. J Am Soc Nephrol. 2014;25(5):1063–1071. doi:10.1681/ASN.2013070742 24357673 PMC4005309

[10] Sarnak MJ Katz R Newman A, . Association of urinary injury biomarkers with mortality and cardiovascular events. J Am Soc Nephrol. 2014;25(7):1545–1553. doi:10.1681/ASN.2013070713 24511130 PMC4073430

[11] Park M Hsu CY Go AS, . Urine kidney injury biomarkers and risks of cardiovascular disease events and all-cause death: the CRIC study. Clin J Am Soc Nephrol. 2017;12(5):761–771. doi:10.2215/CJN.08560816 28254771 PMC5477212

[12] Jin Y Cao JN Wang CX, . High serum YKL-40 level positively correlates with coronary artery disease. Biomark Med. 2017;11(2):133–139. doi:10.2217/bmm-2016-0240 28097894

[13] Blanco-Colio LM Méndez-Barbero N Pello Lázaro AM, . MCP-1 predicts recurrent cardiovascular events in patients with persistent inflammation. J Clin Med. 2021;10(5):1137. doi:10.3390/jcm10051137 33803115 PMC7963189

[14] Amatruda JG Estrella MM Garg AX, . Urine alpha-1-microglobulin levels and acute kidney injury, mortality, and cardiovascular events following cardiac surgery. Am J Nephrol. 2021;52(8):673–683. doi:10.1159/000518240 34515046 PMC8619798

[15] Garimella PS Lee AK Ambrosius WT, . Markers of kidney tubule function and risk of cardiovascular disease events and mortality in the SPRINT trial. Eur Heart J. 2019;40(42):3486–3493. doi:10.1093/eurheartj/ehz392 31257404 PMC6837159

[16] Howard VJ Cushman M Pulley L, . The reasons for geographic and racial differences in stroke study: objectives and design. Neuroepidemiology. 2005;25(3):135–143. doi:10.1159/000086678 15990444

[17] Gillett SR. Validating laboratory results in a national observational cohort study without field centers: the Reasons for Geographic and Racial Differences in Stroke cohort. Clin Biochem. 2014;47(16-17):243–246. doi:10.1016/j.clinbiochem.2014.08.003 25130959 PMC5038129

[18] Halanych JH Shuaib F Parmar G, . Agreement on cause of death between proxies, death certificates, and clinician adjudicators in the Reasons for Geographic and Racial Differences in Stroke (REGARDS) study. Am J Epidemiol. 2011;173(11):1319–1326. doi:10.1093/aje/kwr033 21540327 PMC3101067

[19] Olubowale OT Safford MM Brown TM, . Comparison of expert adjudicated coronary heart disease and cardiovascular disease mortality with the national death index: results from the REasons for geographic and racial differences in stroke (REGARDS) study. J Am Heart Assoc. 2017;6(5):e004966. doi:10.1161/jaha.116.004966 28468785 PMC5524068

[20] Levey AS Stevens LA Schmid CH, . A new equation to estimate glomerular filtration rate. Ann Intern Med. 2009;150(9):604–612. doi:10.7326/0003-4819-150-9-200905050-00006 19414839 PMC2763564

[21] Umapathy D. YKL-40: a biomarker for early nephropathy in type 2 diabetic patients and its association with inflammatory cytokines. Immunobiology. 2018;223(11):718–727. doi:10.1016/j.imbio.2018.07.020 30077474

[22] Rathcke CN. YKL-40, a marker of inflammation and endothelial dysfunction, is elevated in patients with type 1 diabetes and increases with levels of albuminuria. Diabetes Care. 2009;32(2):323–328. doi:10.2337/dc08-1144 18957531 PMC2628702

[23] Schroder J Jakobsen JC Winkel P, . Prognosis and reclassification by YKL-40 in stable coronary artery disease. J Am Heart Assoc. 2020;9(5):e014634. doi:10.1161/jaha.119.014634 32114892 PMC7335588

[24] Kastrup J Johansen JS Winkel P, . High serum YKL-40 concentration is associated with cardiovascular and all-cause mortality in patients with stable coronary artery disease. Eur Heart J. 2009;30(9):1066–1072. doi:10.1093/eurheartj/ehp049 19270316

[25] Ridker PM. Plasma levels of the proinflammatory chitin-binding glycoprotein YKL-40, variation in the chitinase 3-like 1 gene (CHI3L1), and incident cardiovascular events. J Am Heart Assoc. 2014;3(3):e000897. doi:10.1161/jaha.114.000897 24958781 PMC4309091

[26] O'Seaghdha CM. Analysis of a urinary biomarker panel for incident kidney disease and clinical outcomes. J Am Soc Nephrol. 2013;24(11):1880–1888. doi:10.1681/ASN.2013010019 23990678 PMC3810081

[27] Carlsson AC Larsson A Helmersson-Karlqvist J, . Urinary kidney injury molecule-1 and the risk of cardiovascular mortality in elderly men. Clin J Am Soc Nephrol. 2014;9(8):1393–1401. doi:10.2215/CJN.11901113 24923577 PMC4123404

[28] Abdelraheem S. Diagnostic performance of kidney injury molecule-1 for detection of abnormal urinary albumin-to-creatinine ratio in type 2 diabetes mellitus. J Immunoassay Immunochem. 2021;42(6):1954947. doi:10.1080/15321819.2021.1954947 34355651

[29] Lobato GR. Performance of urinary kidney injury molecule-1, neutrophil gelatinase-associated lipocalin, and N-acetyl-beta-D-glucosaminidase to predict chronic kidney disease progression and adverse outcomes. Braz J Med Biol Res. 2017;50(5):e6106. doi:10.1590/1414-431x20176106 28380198 PMC5423741

[30] Peralta CA Katz R Bonventre JV, . Associations of urinary levels of kidney injury molecule 1 (KIM-1) and neutrophil gelatinase-associated lipocalin (NGAL) with kidney function decline in the Multi-Ethnic Study of Atherosclerosis (MESA). Am J Kidney Dis. 2012;60(6):904–911. doi:10.1053/j.ajkd.2012.05.014 22749388 PMC3690926

[31] Damman K Masson S Hillege HL, . Clinical outcome of renal tubular damage in chronic heart failure. Eur Heart J. 2011;32(21):2705–2712. doi:10.1093/eurheartj/ehr190 21666249

[32] Lee AK Katz R Jotwani V, . Distinct dimensions of kidney health and risk of cardiovascular disease, heart failure, and mortality. Hypertension. 2019;74(4):872–879. doi:10.1161/hypertensionaha.119.13339 31378102 PMC6739187

[33] Leiherer A Muendlein A Saely CH, . Serum uromodulin is a predictive biomarker for cardiovascular events and overall mortality in coronary patients. Int J Cardiol. 2017;231:6–12. doi:10.1016/j.ijcard.2016.12.183 28089453

[34] Ahluwalia TS. Uromodulin gene variant is associated with type 2 diabetic nephropathy. J Hypertens. 2011;29(9):1731–1734. doi:10.1097/hjh.0b013e328349de25 21738052

