# Supplementary material for: Urinary Biomarkers of Kidney Tubule Health and Mortality in Persons with CKD and Diabetes Mellitus
Source: Kidney360. 2023 Aug 3;4(9):e1257–64. doi: 10.34067/KID.0000000000000226 (PMC10547219; doi:10.34067/KID.0000000000000226)
Supplement: Supplementary file 1 [file kidney360-4-e1257-s001.pdf]

Supplemental Table 1. Baseline characteristics of the analytical cohort based upon KIM-1 quartiles in REGARDS subcohort.

	KIM-1			
**Quartile (Range)**	**Q1 (< 1016)**	**Q2 (1016 – 1775)**	**Q3 (1776 – 3464)**	**Q4 (> 3464)**
N	**140**	**140**	**140**	**140**
Age, years (SD)	70 (9)	70 (8)	70 (9)	71 (8)
Male sex (%)	61 (44)	69 (49)	65 (46)	68 (49)
Black Race (%)	88 (63)	81 (58)	74 (53)	56 (40)
Education (%)				
Less than high school	29 (21)	30 (21)	34 (24)	30 (21)
High school graduate	31 (22)	38 (27)	40 (29)	43 (31)
Some college	46 (33)	31 (22)	30 (21)	36 (26)
College graduate and above	34 (24)	41 (29)	36 (26)	31 (22)
Insured	137 (98)	135 (96)	134 (96)	136 (97)
BMI	32.2 (6.2)	32.2 (6.7)	31.4 (7.1)	31.5 (6.3)
Hypertension (%)	123 (88)	126 (90)	121 (86)	121 (86)
SBP, mmHg (SD)	130 (18)	134 (21)	135 (19)	133 (17)
DBP, mmHg (SD)	73 (11)	74 (11)	75 (11)	75 (11)
Heart Failure	73 (52)	57 (41)	49 (35)	53 (38)
CAD	55 (39)	46 (33)	63 (45)	61 (44)
Stroke	17 (12)	27 (19)	23 (16)	21 (15)
Smoking				
Never	62 (44)	68 (49)	68 (49)	48 (34)
Former	64 (46)	64 (46)	58 (41)	75 (54)
Current	14 (10)	8 (6)	14 (10)	17 (12)
Antihypertensive medication use	118 (84)	120 (86)	117 (84)	114 (81)
ACE inhibitor/ARB use	107 (76)	103 (74)	109 (78)	96 (69)
Diuretic use	107 (76)	94 (67)	90 (64)	86 (61)
eGFR, mL/min/1.73m^2	41 (13)	42 (12)	40 (13)	39 (12)
UACR, mg/g	18 [7, 62]	37 [10, 124]	39 [10, 336]	71 [15, 789]
<30	87 (62)	67 (48)	64 (46)	52 (37)
30-300	36 (26)	45 (32)	40 (29)	43 (31)
≥300	17 (12)	28 (20)	36 (26)	45 (32)

Abbreviations: angiotensin-converting enzyme (ACE), angiotensin receptor II blocker (ARB) body mass index (BMI), coronary artery disease (CAD), diastolic blood pressure (DBP), estimated glomerular filtration rate (eGFR), standard deviation (SD), systolic blood pressure (SBP), kidney injury molecule (KIM-1), urine albumin-creatinine ratio (UACR).

Supplemental Table 2. Baseline characteristics of the analytical cohort based upon MCP-1 quartiles in REGARDS subcohort.

	MCP-1			
**Quartile (Range)**	**Q1 (< 130)**	**Q2 (130 – 216)**	**Q3 (217 – 385)**	**Q4 (> 385)**
N	**140**	**140**	**140**	**140**
Age, years (SD)	69 (8)	71 (9)	70 (8)	71 (9)
Male sex (%)	61 (44)	80 (57)	61 (44)	61 (44)
Black Race (%)	76 (54)	70 (50)	73 (52)	80 (57)
Education (%)				
Less than high school	31 (22)	28 (20)	32 (23)	32 (23)
High school graduate	36 (26)	28 (20)	38 (27)	50 (36)
Some college	33 (24)	42 (30)	34 (24)	34 (24)
College graduate and above	40 (29)	42 (30)	36 (26)	24 (17)
Insured	138 (99)	134 (96)	138 (99)	132 (94)
BMI	32.4 (6.5)	31.8 (6.6)	31.9 (6.9)	31.4 (6.4)
Hypertension (%)	125 (89)	119 (85)	129 (92)	118 (84)
SBP, mmHg (SD)	131 (17)	131 (16)	133 (19)	137 (23)
DBP, mmHg (SD)	73 (11)	75 (10)	74 (12)	75 (11)
Heart Failure	74 (53)	53 (38)	51 (36)	54 (39)
CAD	54 (39)	60 (43)	57 (41)	54 (39)
Stroke	27 (19)	19 (14)	22 (16)	20 (14)
Smoking				
Never	65 (46)	67 (48)	59 (42)	55 (39)
Former	62 (44)	65 (46)	68 (49)	66 (47)
Current	13 (9)	8 (6)	13 (9)	19 (14)
Antihypertensive use	120 (86)	111 (79)	126 (90)	112 (80)
ACE inhibitor/ARB use	109 (78)	103 (74)	106 (76)	97 (69)
Diuretic use	109 (78)	91 (65)	90 (64)	87 (62)
eGFR, mL/min/1.73m^2	40 (13)	41 (12)	42 (12)	38 (14)
UACR, mg/g	28 [8, 97]	37 [9, 275]	24 [10, 135]	64 [14, 1278]
<30	72 (51)	65 (46)	76 (54)	57 (41)
30-300	54 (39)	41 (29)	35 (26)	34 (24)
≥300	14 (10)	34 (24)	29 (21)	49 (35)

Abbreviations: angiotensin-converting enzyme (ACE), angiotensin receptor II blocker (ARB) body mass index (BMI), coronary artery disease (CAD), diastolic blood pressure (DBP), estimated glomerular filtration rate (eGFR), standard deviation (SD), systolic blood pressure (SBP), monocyte chemoattractant protein-1 (MCP-1), urine albumin-creatinine ratio (UACR).

Supplemental Table 3. Baseline characteristics of the analytical cohort based upon EGF quartiles in REGARDS subcohort.

	EGF			
**Quartile (Range)**	**Q1 (< 767)**	**Q2 (767 – 1017)**	**Q3 (1018 – 1358)**	**Q4 (> 1358)**
N	140	140	141	139
Age, years (SD)	69 (9)	72 (8)	69 (9)	71 (8)
Male sex (%)	74 (53)	69 (49)	59 (42)	61 (44)
Black Race (%)	87 (62)	76 (54)	75 (53)	61 (44)
Education (%)				
Less than high school	28 (20)	37 (26)	31 (22)	27 (19)
High school graduate	49 (35)	38 (27)	33 (23)	32 (23)
Some college	32 (23)	33 (24)	42 (30)	36 (16)
College graduate and above	31 (22)	32 (23)	35 (25)	44 (32)
Insured	133 (95)	135 (96)	137 (97)	137 (99)
BMI	31.7 (6.4)	31.6 (6.8)	32.5 (6.5)	31.7 (6.6)
Hypertension (%)	121 (86)	119 (85)	128 (91)	123 (89)
SBP, mmHg (SD)	138 (23)	133 (18)	132 (16)	129 (17)
DBP, mmHg (SD)	75 (12)	74 (12)	74 (10)	72 (10)
Heart Failure	74 (53)	65 (46)	48 (34)	45 (32)
CAD	66 (47)	56 (40)	52 (37)	51 (37)
Stroke	29 (21)	21 (15)	22 (16)	16 (12)
Smoking				
Never	64 (46)	52 (37)	58 (41)	72 (52)
Former	58 (41)	72 (51)	70 (50)	61 (44)
Current	18 (13)	16 (11)	13 (9)	6 (4)
Antihypertensive use	115 (82)	112 (80)	126 (89)	116 (84)
ACE inhibitor/ARB use	99 (71)	109 (78)	109 (77)	98 (71)
Diuretic use	97 (69)	96 (69)	100 (71)	84 (60)
eGFR, mL/min/1.73m^2	30 (12)	40 (11)	44 (11)	47 (9)
UACR, mg/g	307 [31, 1408]	38 [13, 186]	20 [8, 86]	14 [7, 42]
<30	33 (24)	60 (43)	78 (55)	99 (71)
30-300	36 (26)	50 (36)	47 (33)	31 (22)
≥300	71 (51)	30 (21)	16 (11)	9 (7)

Abbreviations: angiotensin-converting enzyme (ACE), angiotensin receptor II blocker (ARB) body mass index (BMI), coronary artery disease (CAD), diastolic blood pressure (DBP), epidermal growth factor (EGF), estimated glomerular filtration rate (eGFR), standard deviation (SD), systolic blood pressure (SBP), urine albumin-creatinine ratio (UACR).

Supplemental Table 4. Baseline characteristics of the analytical cohort based upon UMOD quartiles in REGARDS subcohort.

	UMOD (pg/ml)			
**Quartile (range)**	**Q1 (< 3080904)**	**Q2 (3080904 – 6197501)**	**Q3 (6197502 – 11294503)**	**Q4 (> 11294503)**
N	140	140	140	140
Age, years (SD)	69 (8)	69 (8)	71 (9)	72 (9)
Male sex (%)	67 (48)	55 (39)	64 (46)	77 (55)
Black Race (%)	76 (54)	77 (55)	76 (54)	70 (50)
Education (%)				
Less than high school	31 (22)	32 (23)	32 (23)	28 (20)
High school graduate	44 (31)	46 (33)	31 (22)	31 (22)
Some college	28 (20)	38 (27)	37 (26)	40 (29)
College graduate and above	37 (26)	24 (17)	40 (29)	41 (29)
Insured	134 (96)	133 (95)	136 (97)	139 (99)
BMI	31.8 (6.6)	32.0 (7.1)	32.3 (6.1)	31.3 (6.4)
Hypertension (%)	125 (89)	127 (91)	121 (86)	118 (84)
SBP, mmHg (SD)	135 (23)	134 (16)	132 (18)	130 (18)
DBP, mmHg (SD)	75 (13)	74 (11)	74 (10)	74 (10)
Heart Failure	77 (55)	65 (46)	40 (29)	50 (36)
CAD	65 (46)	59 (42)	45 (32)	56 (40)
Stroke	22 (16)	30 (21)	19 (14)	17 (12)
Smoking				
Never	68 (49)	61 (44)	57 (41)	60 (43)
Former	59 (42)	62 (44)	70 (50)	70 (50)
Current	13 (9)	17 (12)	13 (9)	10 (7)
Antihypertensive use	117 (84)	122 (87)	115 (82)	115 (82)
ACE inhibitor/ARB use	94 (67)	104 (74)	102 (73)	115 (82)
Diuretic use	98 (70)	102 (73)	88 (63)	89 (64)
eGFR, mL/min/1.73m^2	33 (13)	38 (11)	43 (11)	47 (10)
UACR, mg/g	208 [21, 1272]	33 [10, 155]	25 [9, 125]	18 [7, 51]
<30	44 (31)	67 (48)	73 (52)	86 (61)
30-300	33 (24)	46 (33)	41 (29)	44 (31)
≥300	63 (45)	27 (19)	26 (19)	10 (7)

Abbreviations: angiotensin-converting enzyme (ACE), angiotensin receptor II blocker (ARB) body mass index (BMI), coronary artery disease (CAD), diastolic blood pressure (DBP), estimated glomerular filtration rate (eGFR), standard deviation (SD), systolic blood pressure (SBP), urine albumin-creatinine ratio (UACR), uromodulin (UMOD).

Supplemental Table 5. Baseline characteristics of the analytical cohort based upon a1m quartiles in REGARDS subcohort.

	**a1m**			
**Quartile (Range)**	**Q1 (< 8.25)**	**Q2 (8.25 - 16.60)**	**Q3 (16.61 - 32.10)**	**Q4 (> 32.10)**
N	**140**	**143**	**137**	**140**
Age, years (SD)	70 (9)	71 (9)	71 (8)	69 (8)
Male sex (%)	51 (36)	62 (43)	71 (52)	79 (56)
Black Race (%)	71 (51)	66 (46)	82 (60)	79 (56)
Education (%)				
Less than high school	29 (21)	30 (21)	32 (23)	32 (23)
High school graduate	35 (25)	38 (27)	34 (25)	45 (32)
Some college	35 (25)	39 (27)	37 (27)	32 (23)
College graduate and above	41 (29)	36 (25)	34 (25)	31 (22)
Insured	137 (98)	140 (98)	132 (96)	133 (95)
BMI	33.7 (6.8)	31.7 (6.5)	30.9 (6.7)	31.1 (5.9)
Hypertension (%)	119 (85)	129 (90)	120 (88)	123 (88)
SBP, mmHg (SD)	126 (16)	131 (17)	134 (17)	141 (23)
DBP, mmHg (SD)	71 (10)	72 (9)	75 (11)	79 (12)
Heart Failure	69 (49)	54 (38)	45 (33)	64 (46)
CAD	53 (38)	50 (35)	54 (39)	68 (49)
Stroke	19 (14)	18 (13)	29 (21)	22 (16)
Smoking				
Never	67 (48)	76 (53)	48 (35)	55 (39)
Former	65 (46)	55 (39)	78 (57)	63 (45)
Current	8 (6)	12 (8)	11 (8)	22 (16)
Antihypertensive use	117 (84)	126 (88)	112 (82)	114 (81)
ACE inhibitor/ARB use	112 (80)	110 (77)	102 (75)	91 (65)
Diuretic use	103 (74)	108 (76)	83 (61)	83 (59)
eGFR, mL/min/1.73m^2	45 (11)	44 (12)	41 (12)	32 (13)
UACR, mg/g	12 [5, 35]	21 [9, 64]	45 [13, 220]	443 [45, 2032]
<30	101 (72)	85 (59)	57 (42)	27 (19)
30-300	36 (26)	45 (32)	49 (36)	34 (24)
≥300	3 (2)	13 (9)	31 (23)	79 (56)

Abbreviations: Alpha-1-microglobulin (α1m), angiotensin-converting enzyme (ACE), angiotensin receptor II blocker (ARB) body mass index (BMI), coronary artery disease (CAD), diastolic blood pressure (DBP), estimated glomerular filtration rate (eGFR), standard deviation (SD), systolic blood pressure (SBP), urine albumin-creatinine ratio (UACR)
